# Fabrication and Mechanical Characterization of Dry Three-Dimensional Warp Interlock Para-Aramid Woven Fabrics: Experimental Methods toward Applications in Composite Reinforcement and Soft Body Armor

**DOI:** 10.3390/ma13194233

**Published:** 2020-09-23

**Authors:** Mulat Alubel Abtew, Francois Boussu, Pascal Bruniaux, Han Liu

**Affiliations:** 1 College of Textile and Clothing Engineering, Soochow University, 178 G.J.D. Road, Suzhou 215021, China; mulat-alubel.abtew@ensait.fr; 2 Faculty of Science and Technology, University of Lille, Nord de France, 59000 Lille, France; Francois.boussu@ensait.fr (F.B.); pascal.bruniaux@ensait.fr (P.B.); 3 GEMTEX Laboratory, ENSAIT, 2 Allée Louise et Victor Champier, 59056 Roubaix, France; 4 Faculty of Textiles—Leather Engineering and Industrial Management, Gheorghe Asachi’ Technical University of Iasi, Dimitrie Mangeron Bd. 53, 700050 Iasi, Romania

**Keywords:** 3D warp interlock fabric, warp yarn interchange ratio, mechanical test, mechanical characterization, fiber-reinforced composite, soft body armor, para-aramid fiber

## Abstract

Recently, three-dimensional (3D) warp interlock fabric has been involved in composite reinforcement and soft ballistic material due to its great moldability, improved impact energy-absorbing capacity, and good intra-ply resistance to delamination behaviors. However, understanding the effects of different parameters of the fabric on its mechanical behavior is necessary before the final application. The fabric architecture and its internal yarn composition are among the common influencing parameters. The current research aims to explore the effects of the warp yarn interchange ratio in the 3D warp interlock para-aramid architecture on its mechanical behavior. Thus, four 3D warp interlock variants with different warp (binding and stuffer) yarn ratios but similar architecture and structural characteristics were engineered and manufactured. Tensile and flexural rigidity mechanical tests were carried out at macro- and meso-scale according to standard EN ISO 13 934-1 and nonwoven bending length (WSP 90.5(05)), respectively. Based on the results, the warp yarn interchange ratio in the structure revealed strong influences on the tensile properties of the fabric at both the yarn and final fabric stages. Moreover, the bending stiffness of the different structures showed significant variation in both the warp and weft directions. Thus, the interchange rations of stuffer and binding warp yarn inside the 3D warp interlock fabric were found to be very key in optimizing the mechanical performance of the fabric for final applications.

## 1. Introduction

Textile materials nowadays are widely used in various technical applications, including composite reinforcement for aerospace, transport, military, and other applications. The structure of the fabric involved in technical applications should have higher mechanical performance compared to the structure involved in conventional applications such as clothing and home furnishings. Previously, two-dimensional (2D) woven (in the form of plain, twill, and satin) and unidirectional (UD) fabric structures were mainly used and discussed regarding their mechanical performance under different loading conditions [1,2]. Apart from 2D and UD fabrics, three-dimensional (3D) woven fabric have also become promising for use in composite materials, such as fibrous reinforcement and protective solutions, as well as other technical applications [3,4]. Its involvement in the above application is mainly because it has an improved capacity to absorb energy by higher intra-ply resistance to delamination, saves on cost, and has high production rates [5,6,7]. Moreover, such fabric also provides better shaping ability for 3D shape solutions by linking different yarns through different weave styles at the required thickness [8,9,10,11]. However, its mechanical properties are much different from those of conventional 2D woven and unidirectional fabric due to its complex fabric structure. This has attracted the attention of various researchers to understand the mechanical behavior before recommending particular applications. Based on this, various researchers have been intensively investigating not only the final mechanical behavior under static or dynamic loading at the dry and composite reinforcement stages, but also the influence of various factors, including fabric type, fabric architecture, fiber type, yarn density, and weave parameters [12,13,14].

The fabric weave type and structure are among the most important factors that greatly affect the mechanical properties of dry 3D warp interlock fabrics [15,16,17,18,19,20]. In addition, detailed experimental observations of 3D woven composites have indicated that the geometry of the fabric has a dominant role in determining the mechanical properties and associated failure mechanisms [21]. Another interesting feature of 3D warp interlock fabric is that it not only provides room for managing different weft layer parameters inside the fabric architecture, but also allows relatively accurate control of the yarn’s evolution in the structure [22]. A research work experimentally studied the effect of the fabric structure on the tensile behavior of 3D woven composite using four 3D woven multilayer fabric structures as reinforcement [16]. According to the investigation, the fabric structure showed a great effect not only on the tensile strength but also on the dimensional stability of the composites. Similarly, another study also supported the effect of fabric structure on the overall mechanical properties of 3D textile composites [17]. The effect of fabric type on the mechanical behavior of reinforced composites using unidirectional (UD), two-dimensional (2D), three-dimensional (3D) orthogonal, 3D angle-interlock, and 3D warp interlock multilayers as reinforcement was also comprehensively studied. The results showed that composites made with 3D woven fabric had considerably better impact resistance, knife penetration resistance, and dynamic mechanical analysis (DMA) behavior as compared with their UD and 2D counterparts [18].

Apart from the type of weave architecture and raw material type (fiber and yarn), warp yarn crimp values in the woven structure and their combinations (hybridization) also affect the mechanical behavior of both dry 3D warp interlock fabrics and composites [23,24,25,26]. A study on the tensile properties of 3D woven reinforced composite made of Kevlar fiber revealed higher tensile strength as compared to nylon-reinforced composite. Moreover, a hybridized composite made of both Kevlar–glass and Kevlar–glass–nylon fibers showed great improvement in tensile strength as compared to a monolithic composite made of glass fiber [24]. The effect of fiber type and hybridization on the mechanical properties of three-dimensional angle-interlock composite fabrics has also been investigated. Bandaru et al. [25] investigated the effect of fabric hybridization on the mechanical behavior. For this, two types of fabrics, one made of Kevlar and basalt yarns individually and another made of a combination of Kevlar and basalt yarns, were developed and tested with the quasi-static tensile test. Based on the results, hybridization improved the tensile behavior of the three-dimensional angle-interlock composite fabric. The weaving process and its parameters, including the positioning of stuffer, fiber volume fractions, and linking warp yarns in the 3D orthogonal and warp interlock woven fabric structure, were also shown to have an influence on the mechanical properties of the fabrics [27,28,29].

In addition, yarn and weave densities also play an essential role in the mechanical properties of 3D woven fabrics [30,31]. To validate this, an experimental study was conducted on 3D layer-to-layer glass/epoxy woven composite structures made with different pick densities. In general, the study revealed that reducing the waviness and misalignment of load-carrying fibers improved the mechanical properties of the material [30]. Nasrun et al. [32] investigated the effects of weft density on the mechanical tensile strength behavior of 3D angle interlock woven fabric. Four fabrics based on different weft densities (12, 16, 20, and 24 picks per cm) using polyester plied yarn with 100 Tex were produced and tested based on ASTM standards. According to the results, the tensile strength of 3D angle interlock woven fabric could be improved with increased weft density. The effect of Z-yarn on the mechanical properties of 3D weave architecture was also studied and discussed based on the fabric geometry and 3D finite element simulations [33]. An interlocking pattern is another factor that affects the final mechanical performance of three-dimensional orthogonal layer-to-layer interlock composites. Three types of orthogonal layer-to-layer interlock fabrics—warp, weft, and bi-directional interlock composite—were tested for their mechanical performance [34]. Due to their lower crimps, the warp and weft interlock composites showed better tensile behavior as compared to the bi-directional interlock composite. The off-axis angles among the different yarns while developing the 3D woven fabrics also showed an influence on the mechanical properties of the materials [35,36].

Apart from the influencing parameters described above, the current study investigates and discusses the effect of the warp yarn interchange ratio on the mechanical behavior of 3D warp interlock fabrics. We designed, manufactured, and experimentally investigated the mechanical behaviors of 3D warp interlock para-aramid fabrics under quasi-static conditions. For this investigation, four 3D warp interlock para-aramid fabric architectures considering the binding–stuffer warp yarn interchange ratio were designed and fabricated. All designed orthogonal layer-to-layer (O-L) fabric architectures were manufactured considering the same warp and weft yarn densities. The test program includes uniaxial yarn tensile tests, flexural rigidity tests, and a uniaxial fabric tensile test. Characterization of high-performance 3D warp interlock para-aramid fabrics provides an understanding for different technical applications, including ballistics, for better performance.

## 2. Materials and Methods

### 2.1. Materials

**Figure 1:**
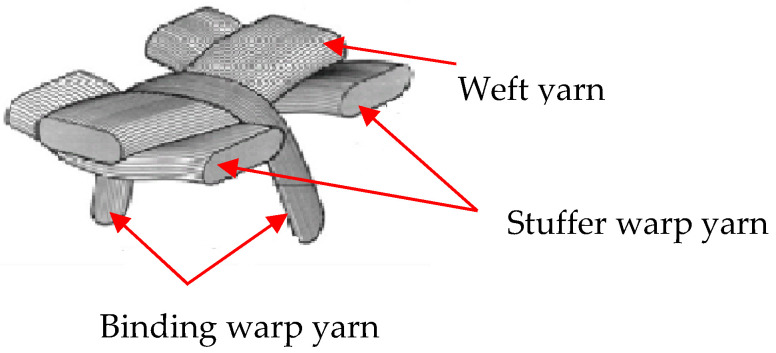
General schematic diagram of deformations of binding, stuffer, and weft yarns in three-dimensional (3D) woven fabric [38].

The arrangement, movement, and deformational behavior of different warp (stuffer and binding) and weft yarns can affect the overall properties of fabric. According to Reference [37], stuffer and binding warp yarns can define the in-plane and through-thickness properties respectively, of the final fabric, whereas weft yarn also helps to define the number of fabric layers as well as determine the transverse properties of the fabric. Figure 1 shows the general interlacing structure of the different yarns in 3D woven fabric. To understand the effects of the warp yarn interchange ratio on the fabric’s mechanical behaviors, four 3D warp interlock fabric architectures based on different binding and stuffer warp yarn interchange ratios were designed and manufactured.

**Figure 2:**
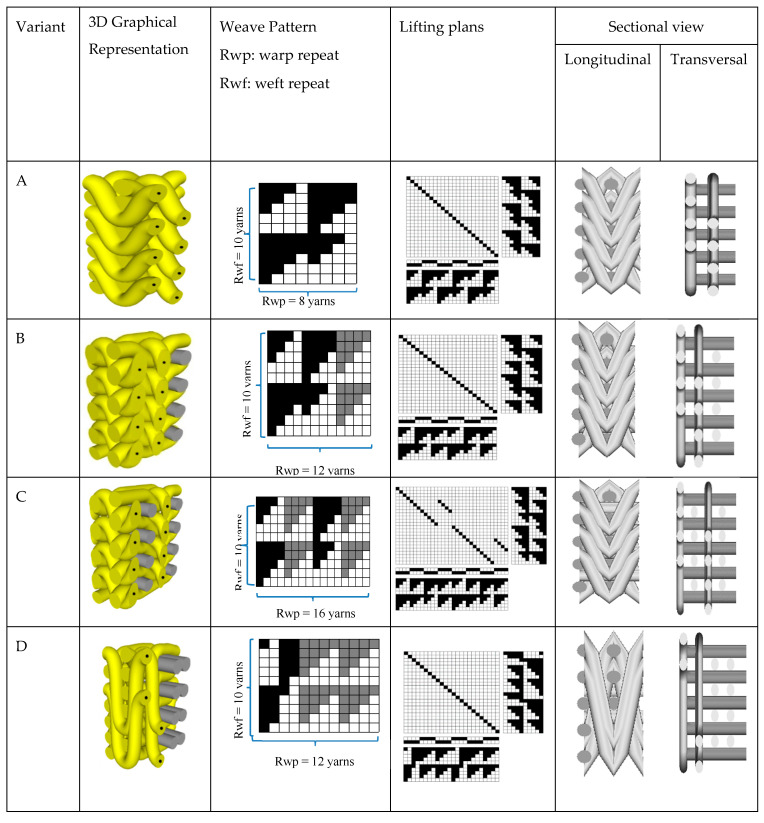
Three-dimensional (3D) graphic representation and weave pattern of repeat unit for 3D warp interlock fabric with different interchanging ratios between binding and stuffer warp yarn.

All of the 3D warp interlock fabric architectures were manufactured using high-performance 930dtex p-aramid fibers (Twaron^®^ f1000), delivered by Teijin Aramid (Wuppertal-Elberfeld, Germany), a subsidiary of the Teijin Group, the Netherlands in ENSAIT-GEMTEX Laboratory, Roubaix. Such fiber is used to produce the most recommended 2D woven fabrics (Twaron CT-709) by the Teijin Company for the development of body armor due to its good ballistic performance and high level of molding capability. The 3D warp interlock fabric architectures were designed with different binding and stuffer warp yarn interchange proportions in the fabric repeat unit: variant A: 100% binding, 0% stuffer, variant B: 66.7% binding, 33.3% stuffer, variant C: 50% binding, 50% stuffer, and variant D: 33.3% binding, 66.7% stuffer, as shown in Figure 2.

Unlike the warp yarn composition in the structure and the fabric thickness, all developed 3D warp interlock fabrics were designed as orthogonal layer-to-layer (O-L) and had the same fiber type, number of weft layers, and yarn density. High-performance fibers with 2.35 mN/Tex of tenacity, 225 N strength at break, and 3.45% of elongation at break were used for all the fabrics. Each multilayer 3D warp interlock fabric also involved five weft layers. Also, 48 ends/cm/panel and 50 picks/cm/panel yarn densities were used in the warp and weft directions respectively, when manufacturing the fabrics. The theoretical fabric weight was computed as 970 g/m^2^ for all fabric structures. The average fabric thickness was measured as 1.42, 1.44, 1.52, and 1.63 mm for variants A, B, C, and D, respectively. Such thickness variation among fabrics arises due to the interlacing behavior of each weft layer by the different binding warp yarns.

**Figure 3:**
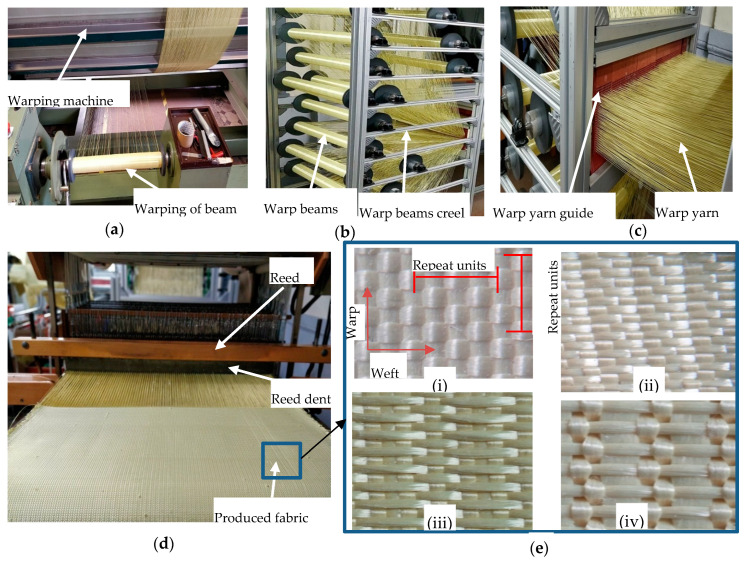
Manufacturing process of 3D warp interlock fabric using semi-automatic loom: (**a**) warping process and winding of warp ends to warp beam, (**b**) warp beam arranged in adapted warp beam creel, (**c**) warp yarn guiding system, (**d**) weaving machine head and weaving process, and (**e**) top surface view of produced 3D warp interlock fabrics ((**i**) variant A, (**ii**) variant B, (**iii**) variant C, (**iv**) variant D).

Commercially available software, TexGen^®^ and WiseTex^®^, were used to develop the 3D geometric and weave peg plans of the fabrics, respectively. The fabricated multilayer fabrics were then woven by using a modified semi-automatic ARM dobby loom. The ARM dobby loom (with a 50 cm width) was designed with an adapted warp beam creel to properly accommodate up to 24 warp beams and can produce all kinds of 3D warp interlock fabrics. It also has a yarn guiding system to separate, accommodate, and guide warp yarn ends before weaving head sections, as shown in Figure 3.

### 2.2. Experimental Testing Methods

In this section, different testing methodologies and procedures for experimental testing, measuring, and characterizing the produced 3D warp interlock fabrics will be explained. Uniaxial tensile tests on different types of yarn in the fabric (Manufactured by Instron, Norwood, MA, USA and having two clamps initially 200 mm apart and a speed of 100 mm/min at room temperature), flexural rigidity tests (using Cantilever customized in GEMTEX Laboratory, Roubaix, France), and uniaxial fabric tensile tests (Manufactured by Instron, Norwood, MA, USA) were performed to characterize and understand the effects of the binding:stuffer yarn interchange ratio on the mechanical properties of 3D warp high-performance interlock p-aramid fabrics. Moreover, various fabric properties including actual thickness (mm) and density (g/m^2^) were computed. The average actual fabric thickness and weight of the 3D warp interlock fabrics were precisely measured according to NF EN ISO 5084 [39] and NF EN 12127 [40] standards, respectively. The top and cross-sectional views of the produced 3D warp interlock fabrics were examined using a portable optical microscope (dnt professional liquid crystal display (LCD) digital microscope portable camera equipped with Universal Serial Bus (USB)/Thin Film Transistor (TFT) 5 MPix zoom 20 to 500× dnt DigiMicro Lab 5.0). Figure 3e shows the top views of the 3D warp interlock p-aramid fabrics.

#### 2.2.1. Yarn and Fabric Uniaxial Tensile Testing Setup and Procedure

For textile materials that are intended for use in various applications that demand high mechanical behavior (e.g., composite reinforcement and soft ballistic material), we need a better understanding of their behavior at different levels. Thus, investigating the mechanical behavior of the material at the yarn level greatly helps in determining the yarn mechanics in applications at both dry and composite stages. For example, yarn density, modulus of elasticity and deformation, yarn strain and stress on impact, yarn-to-yarn friction, yarn tenacity, etc., are some of the critical properties that affect the material’s ballistic performance [41,42,43]. In this section, 10 samples of p-aramid yarns from each 3D warp interlock fabric structure, considering the warp (stuffer and binding) and weft (different weft layers) directions, were carefully drawn without damaging the fibers/filaments to investigate and understand the mechanical behavior.

**Figure 4:**
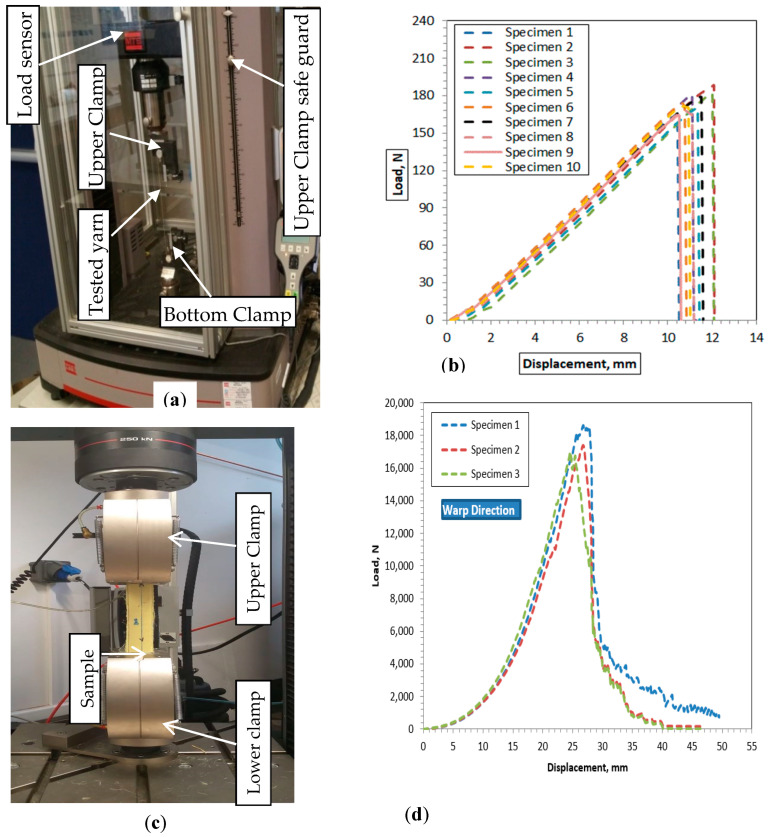
Uniaxial tensile testing: (**a**) yarn tensile testing device setup, (**b**) examples of uniaxial warp-binding tensile test results for sample fabric D in the machine direction (MD), (**c**) fabric tensile testing device setup, and (**d**) examples of uniaxial fabric tensile test results of samples in the machine (warp) direction for variant A.

The uniaxial tensile test helps in investigating parameters including the stress–strain relationship, elastic modulus (E), maximum stress (σmax), and maximum strain (εmax) values of the yarn in both the machine (warp) and cross (weft) directions. In our investigation, the uniaxial tensile tests were conducted using an Instron 8516 universal testing machine manufactured by Instron (Norwood, MA, USA) with a 5 KN load cell at a velocity of 50 mm/min, as shown in Figure 4a. To guarantee repeatability of the results, the entire yarn test was performed with 10 replicas for each sample in both the warp and weft directions (Figure 4b). Each yarn sample was prepared with a total length of 250 mm and firmly fixed at both ends at 200 mm distance using two adapted steel clamps to avoid any slippage while testing. The fabric sample tensile behavior was also performed on the Instron 5900 testing machine with a 250 KN load cell according to EN ISO 13 934-1 standard [44], as shown in Figure 4d. A rectangular 300 × 50 mm sample that was adhesively bonded to 50 mm using resin at both ends was prepared to avoid slippage between the sample and the clamp jaw. Before every test, the two separate jaws (movable top clamp and fixed lower jaw) of the testing machine were set at a distance of 200 mm between them. The sample was firmly mounted between the upper and lower clamps to coincide with the resin bonded size to avoid any load displacement reading errors due to slippage between the sample and the clamp. Three samples for each fabric type were tested at 100 mm/min in the weft and warp directions to ensure repeatability of the investigation (Figure 4b). For each test, forces vs. deformation values with time duration were automatically recorded for all samples. Moreover, the tensile testing machine was also checked after each test so as to achieve accurate results by avoiding slippage between the sample and the clamp. The average tensile properties of the samples in the weft and warp directions were then analyzed using the extracted data during the test. To determine the effect of the warp yarn ratio on the breaking strength and elongation of 3D warp interlock fabrics, the measurement results obtained from the tests were analyzed and evaluated.

#### 2.2.2. Flexural Rigidity Test of Fabrics

**Figure 5:**
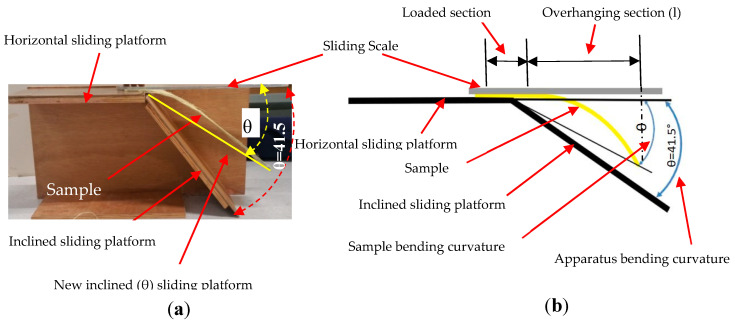
(**a**) Photograph and (**b**) schematic of flexural rigidity test of 3D warp interlock fabrics in stiffness testing apparatus.

The effects of stuffer and binding warp yarn interchange ratio on the flexural rigidity properties of the 3D warp interlock fabrics were investigated. A bending test under mass was performed using a fabric stiffness testing apparatus following the Standard Test Methods for Nonwoven Bending Length (WSP 90.5(05)) under the principle of the cantilever. For this test, 300 × 50 mm^2^ rectangular strips of samples for the warp and weft directions were prepared. Before testing, the samples were kept in standard atmosphere conditions (relative humidity (RH) 65% ± 2% and temperature 20 ± 2 °C) to bring them to moisture equilibrium as directed by ERT 60.2-99 and ISO 554. Figure 5 shows pictorial and schematic illustrations of the fabric stiffness testing apparatus while testing the flexural rigidity properties of a sample. The apparatus was designed with bending curvature according to ISO 4604 with a fixed angle (41.5°) and its platform was set up to accommodate proper samples while testing. Five samples in both directions (weft and warp) were examined for each fabric type, and the bending length was computed based on the average values. All samples were weighed using a digital balance with approximately ±0.001 g precision based on TS 251 before every test.

During testing, the sample was properly positioned on the horizontal platform, on which one edge was fixed and the other was free to hang on the platform. The sample was allowed to slide on the horizontal sliding platform by pushing gently at a regular rate using a regular sliding scale. In our case, the investigation to compute flexural rigidity was performed in two ways. Some tests were performed until the sample was overhanging by its weight and the sample edges at the front touched the inclined sliding platform (41.5°). In other tests, if the overhanging sample did not touch the inclined platform, the overhanging sample (l) and the sample bending curvature (θ) were measured for further computation. For both cases, the flexural rigidity of the sample was then computed considering overhanging length (l), bending length, and the sample’s areal weight and bending curvature. The flexural bending rigidity (N.m) was computed based on Equation (1):

(1) G=1tanθCosθ2×ρ×l38

where *G* is fabric flexural bending rigidity, *ρ* is fabric sample weight per unit area (mass per unit area × gravitational acceleration), l is overhanging length, and θ is bending curvature.

In general, the average bending length of the samples can be calculated using Equation (2):

(2) C= l2

where *C* is the sample bending length and l is the sample overhanging length after the bending test.

When the front edges of the sample touched the inclined sliding platform (41.5°) of the apparatus, the fabric flexural bending rigidity could be calculated by Equation (3):

(3) G=1tanθCosθ2 × ρl38, for θ = 41.5°, 1 tanθcosθ2=1

Here, $G=1\;\times \;\frac{\rho l^{3}}{8}\;$, since l/2 is the bending length and *ρ* is mass per unit area multiplied by acceleration due to gravity, and the fabric flexural bending rigidity can be simplified as follows (Equation (4)):

(4) $$G=\;W\;\times \;g\;\times {\;c\;}^{3}$$

where G is the flexural bending rigidity of the sample (N m), W is the sample unit areal weight (g/m^2^), C is the average bending length of the sample (mm), and g is gravitational acceleration (m/s^2^).

## 3. Results and Discussion

In this section, the mechanical behavior of the developed 3D warp interlock fabrics considering different binding and stuffer warp yarn compositions is discussed, and the yarns in the 3D fabric structure are investigated and discussed. The fabric and its corresponding yarn tensile behavior were evaluated based on the stress–strain response curve and the average maximum tensile stress (MPa) and strain (%) values at the fracture point. The rigidity behavior of the fabrics was also assessed based on flexural bending rigidity (N.m) and fabric bending length. Finally, the effects of warp yarn composition on the waviness of the yarn in the 3D warp interlock fabrics in both directions were explained in terms of crimp percentage.

### 3.1. Yarn Uniaxial Tensile Property

A yarn’s tensile property can be defined as the maximum applied force/load that is required to break the yarn. Understanding this property is very important not only because it is a key parameter for the fabrication of yarn, but it directly influences the strength of the developed fabrics.

#### 3.1.1. Stuffer and Binding Warp Yarn Testing

**Figure 6:**
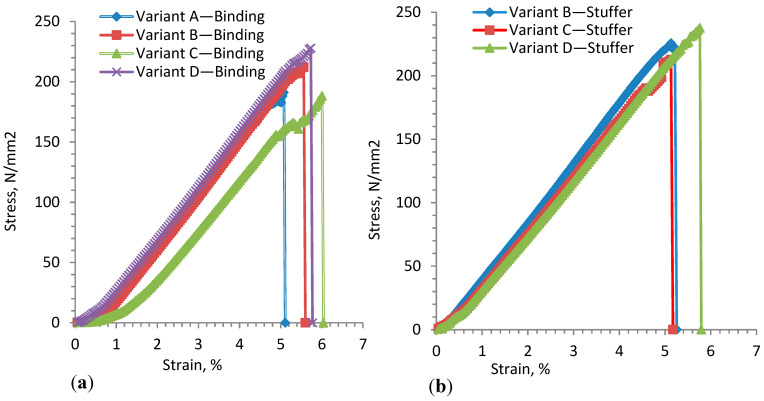
Typical stress–strain curves of (**a**) warp-binding yarn and (**b**) warp-stuffer yarn for different 3D warp interlock fabric architecture.

The 3D warp interlock fabric structure not only has weft and warp yarns in the plane direction, but also another warp yarn type through the thickness direction [37]. This reinforces the fabric in three directions, and each group of yarns provides a specific function to the structure. The composition of one or more types of warp yarn mainly depends on the final application. The 3D warp interlock fabric can be represented with different weft layers of fabric interlaced through the thickness direction by using binding warp yarns. Thus, the fabric could present different mechanical behaviors due to different interlacement and binding positions of the yarns. Figure 6a,b shows the average stress–strain curves for warp-binding and warp-stuffer yarn for the 3D warp interlock fabrics. Based on the stress–strain curves, it is also possible to analyze the mechanical properties of each yarn in the fabric. The tensile stress–strain curve shows similar trends for the binding and stuffer yarn in the fabrics. As observed in Figure 6a, except for variant C, the higher the proportion of binding warp yarn in the fabric, the lower the tensile modulus (E) in the warp direction. The average tensile modulus (E) of binding warp yarn for variant D was found to be higher compared to the other samples. Variant C showed the lowest tensile modulus (E), followed by B and D.

This is because the binding warp yarn in variant C occupies a lesser interlacement depth along with higher stuffer yarn to bind the wet layers. As shown in Figure 6b, a very similar tensile modulus (E) of the stuffer yarn was observed for all fabrics. However, the tensile modulus of the stuffer yarn was found to be a little bit higher for samples with a smaller proportion of stuffer yarn, and vice versa. Variant B shows a higher tensile modulus, whereas variant D recorded the lowest. Table 1 shows the tensile stress and strain at failure for binding and stuffer warp yarns of the developed variants.

**Figure 7:**
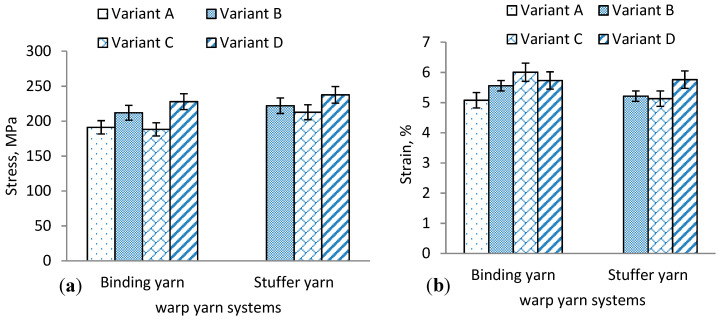
(**a**) Average maximum tensile stress and (**b**) tensile failure strain of tested sample for binding and stuffer warp yarns for different fabrics.

Figure 7a,b also shows the average maximum stress at failure (σmax) and tensile failure strain (εmax) for the respective warp yarns. The maximum stress is higher in the case of fill yarns; however, the failure strain is quite similar in both directions. The average maximum stress and tensile strain at failure for binding warp yarn of variant A showed lower values than other fabric types. The maximum tensile failure strain of binding warp yarn was obtained for variant C, followed by variant D, whereas the maximum failure stress of binding warp yarn was obtained for variant D. The elongation failure of binding warp yarn could be affected by the occurrence of filament degradation due to friction between the yarns while linking the weft layers in the weaving process more than stuffer warp yarn. However, unlike the binding yarn, the maximum stress and tensile strain at failure of stuffer yarn show a similar trend in all samples. Both stress and strain at failure were higher for variant D, followed by variants B and C.

#### 3.1.2. Weft Layer Yarn Testing

**Figure 8:**
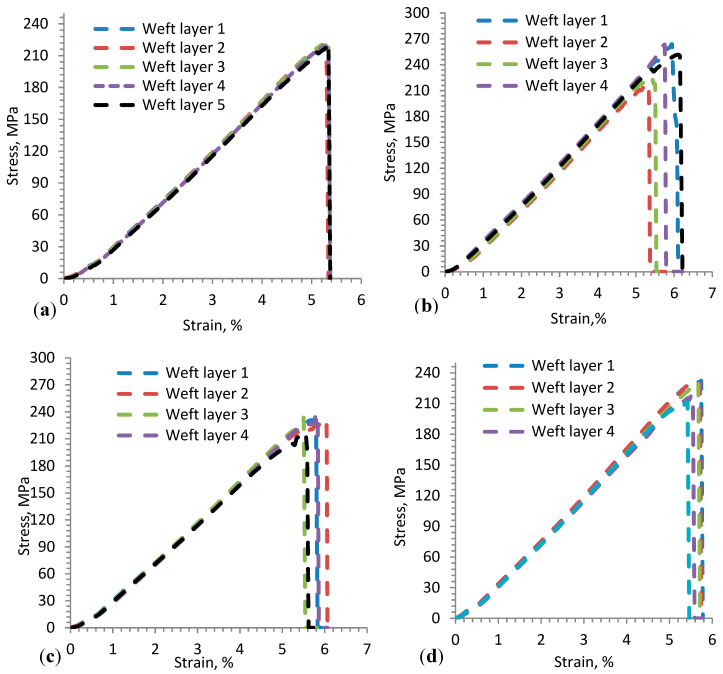
Typical stress–strain curves of layers 1–5 of weft yarn for (**a**) variant A, (**b**) variant B, (**c**) variant C, and (**d**) variant D 3D warp interlock fabric architecture.

The tensile behaviors of weft yarn in the 3D warp interlock fabrics can be influenced by various parameters. In Figure 8a–d, the tensile stress–strain behaviors of weft layer yarns of 3D warp interlock fabrics are shown to generally have more or less a similar trend. However, both the binding and stuffer warp yarn interchange ratio and the location of weft yarn in the weft layer can influence the tensile properties of the corresponding weft yarn. The fabric with the highest or lowest proportion of binding warp yarn exhibited no effect on the final weft yarn tensile properties. The tensile stress and strain at failure for weft layer yarns of the developed variants are summarized in Table 2.

For example, variants A and D show approximately similar tensile strength (E) of weft yarn at failure within the respective layers as compared to variants C and B. Moreover, even though the positions of weft yarn in the fabric layer affects its tensile properties, clear trends were not observed for the fabric variants.

**Figure 9:**
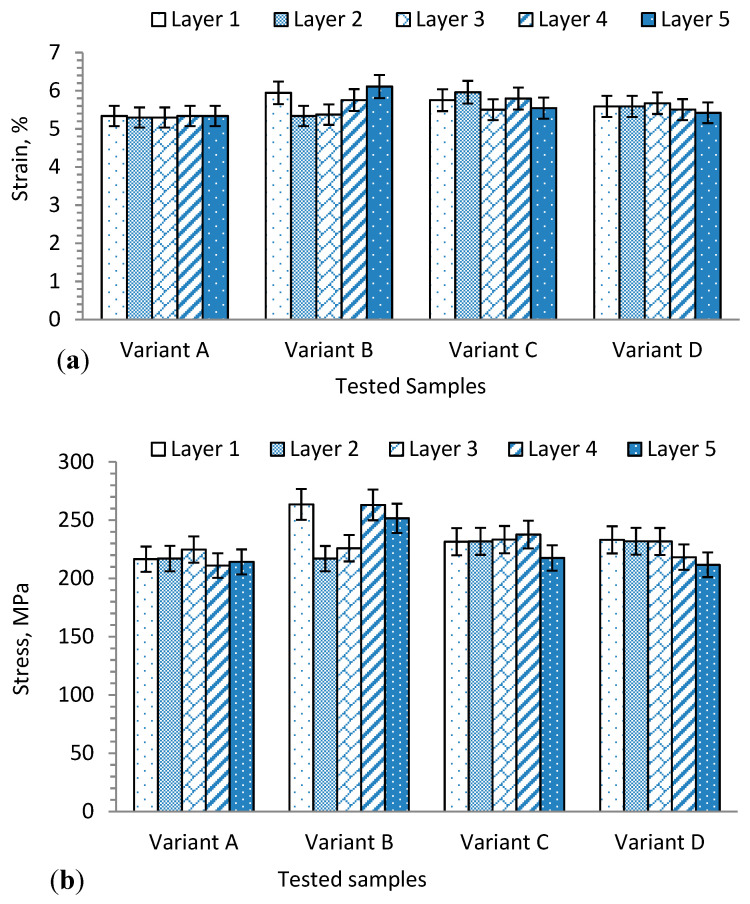
(**a**) Average maximum tensile failure strain and (**b**) maximum tensile failure stress of tested sample for weft yarns in different layers.

Moreover, based on the stress–strain curves, the mechanical properties of the 3D warp interlock fabric samples were characterized based on their unique tensile behavior of stress (MPa) and corresponding strain (%) at the breaking point. Figure 9a,b shows the average maximum stress (MPa) and strain (%) values at the fracture point for the samples in the warp and weft directions. These values were found to be more or less similar in variants A and D compared to variants B and C. Weft yarn in layer 1 shows maximum load and tensile strain (%) in variant B as compared to the majority of weft layers, whereas weft yarn in layer 5 shows lower maximum tensile strain (%) than other weft layers.

### 3.2. Fabric Uniaxial Tensile Property

**Figure 10:**
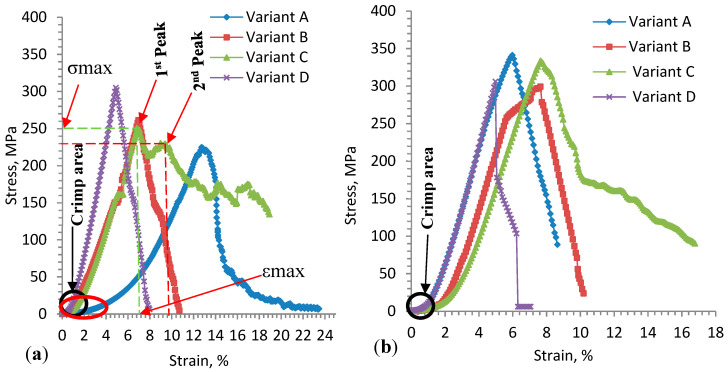
Typical stress–strain curves of 3D warp interlock fabric architectures in (**a**) warp (machine) direction, and (**b**) weft (cross) direction.

Uniaxial tensile stress vs. tensile strain of the 3D warp interlock orthogonal layer-to-layer fabrics made with different warp yarn ratios was experimentally examined in the warp and weft directions. In this section, these test results will be discussed. The stress (MPa) vs. strain (%) curves of the three tensile tests for 3D warp interlock fabric samples are described in Figure 10a,b. The test was performed until the sample reached the maximum deformation state and failed. In general, the stress–strain curve of the samples indicates a similar progression, where the tensile stress values become higher as the strain value increases. The stress–strain curve comprises three main parts: the first section shows a nonlinear curve (crimp area), the second section shows higher stress vs. strain values (elongation area), and the last section shows declining stress–strain values. In the first section, the crimping area at the beginning of the curve shows almost negligible values in the applied loading direction and deformation in the displacement direction at the beginning, and then gradually increases. This is mainly due to the straightening of yarn in the fabric before real deformation occurs along the load direction. When it is closely observed, the crimp area (marked with a black circle) was found to be similar for all samples in the weft direction.

However, due to the different warp yarn (binding and stuffer yarn) composition in the various fabrics, all samples showed different crimp areas in the warp direction (black and red). For example, variant A shows more straightening area (marked with a red circle) as compared to the other samples in the warp direction, whereas variant D shows a higher crimp area, followed by variants B and C. The maximum strain absorbed in the crimp area for weft and warp directions was approximately 1.13% and 1.72%, respectively.

The second section of the stress–strain curve (Figure 10a,b) shows different linear progression with the rapid growth of tensile stress and strain until the sample reached its breaking point in warp and weft directions. The tensile stress vs. strain curve shows more or less a similar trend for all samples in the weft direction due to the balanced weft yarn composition in the fabric. Even though the curve is linearly progressive, the different warp yarn system leads to varying tensile stress and strain relationships in the warp direction. Most obviously, due to the balanced proportions of stuffer and binding warp yarns in the fabric, the typical stress–strain curve of variant C displays two main peak points in the warp direction (Figure 10a). The dominant first and second failure peaks were due to the failure of stuffer and binding warp yarns, respectively. Such condition arises mainly from the actual length difference between stuffer and binding warp yarns in the fabric while loading. On the contrary, different peak points cannot be seen for the other samples with either the same or one dominant warp yarn (stuffer or binding) system in the warp direction. For instance, variant A (100% binding warp yarn) shows a gentler progressive slope and single tensile failure peak with lower tensile modulus (E) in the warp direction, as shown in Figure 10a, whereas variant D (66.7% stuffer warp yarn) demonstrates a steeper linear progressive curve with a single peak and higher tensile modulus (E). The higher tensile modulus value of variant D is due to its higher stiffness behavior, which comes from the higher proportion of stuffer warp yarn in the fabric in the warp direction, whereas the lower value for variant A is due to the higher waviness of the binding warp yarn in the fabric, which needs more load to deform the sample. Variants B and C lie in between and show similar linear progressive slope with approximately equal tensile modulus (E) until the breaking peak point in the warp direction (Figure 10a).

**Figure 11:**
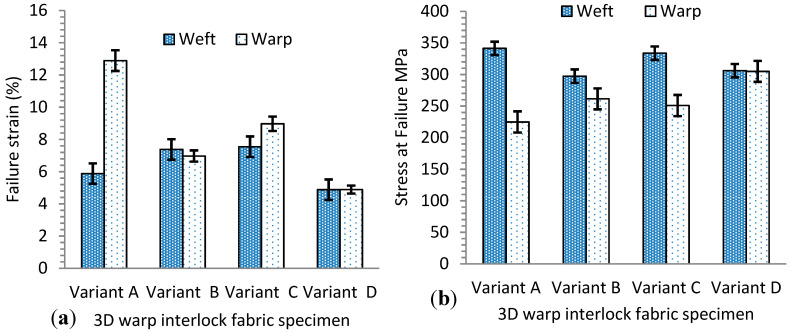
(**a**) Average maximum tensile strain (εmax), and (**b**) tensile stress (σmax) at failure for 3D warp interlock fabric samples in warp and weft directions.

Unlike the warp direction, the stress vs. strain of fabrics in the weft direction show almost linear and elastic progressive curves with approximately similar trends (Figure 10b). This might be because they have the same weft yarn composition and density among all fabric in the weft direction. However, a pressing effect of the warp yarn on the weft yarn during the weaving process could affect the slope of the curve and tensile strength values. Variants A and C show approximately similar tensile stress (E), with higher values of 341.40 ± 10 MPa and 333.82 ± 12 MPa respectively, as compared to variants B (297.31 ± 9.8 MPa) and D (306.10 ± 8.2 MPa). The tensile stress and strain at failure for the variants are summarized in Table 3. Here, the value of each sample is the average of three samples tested. This is due to the presence of higher weft yarn undulations in variants A and D, which can absorb more exerting forces in the loading direction. Variant B also recorded better tensile modulus (E) as compared to variant C. Even though the 3D warp interlock fabrics have the same structure and areal density, they show different maximum tensile strain (εmax) and stress (σmax) at failure. Figure 11 shows the fabric samples’ tensile strain and strength at failure in the warp and weft direction.

Considering the weft direction, the fabric samples did not show a significant difference in maximum tensile strain (εmax) and stress (σmax) at failure, as shown in Figure 11a,b. This is because they had the same weft yarn composition in their 3D architecture. The slight difference arises from the stressing effects of warp yarn while interlacing to form the fabric. For example, variants A and B failed at tensile strain values around 5.88% ± 0.68% and 7.38% ± 0.9% when the stress was applied along the weft direction, whereas variants C and D failed at strain values of 7.55% ± 0.69% and 4.88% ± 0.76%. However, due to the different warp yarn proportions in the fabric architecture, the maximum tensile strain (εmax) and stress (σmax) at failure were found to be different in the warp direction in the 3D warp interlock fabric samples. For example, variant A (100% binder warp yarn) showed higher tensile strain at failure in the warp direction (12.88% ± 1.1%) than the weft direction (5.88% ± 0.68%) due to the higher undulating property of binder warp yarn in the fabric architecture. On the contrary, variant D (66.7% stuffer, 33.3% binding warp yarn) showed nearly equal tensile strain at failure in both the warp and weft direction (4.89% ± 0.64% and 4.88% ± 0.76%). This is because both directions have approximately equal undulation of warp and weft yarns in the fabric structure. Similarly, the tensile stress at failure for the fabric samples was also affected by the warp yarn composition in the fabric structure. For example, the tensile responses of variant D were found to be equal in the warp and weft directions due to the approximately similar undulation behavior of yarn in both directions, whereas variants A, B, and C showed different tensile stress at failure in the warp and weft directions due to their various binding warp yarn composition (Figure 11b).

**Figure 12:**
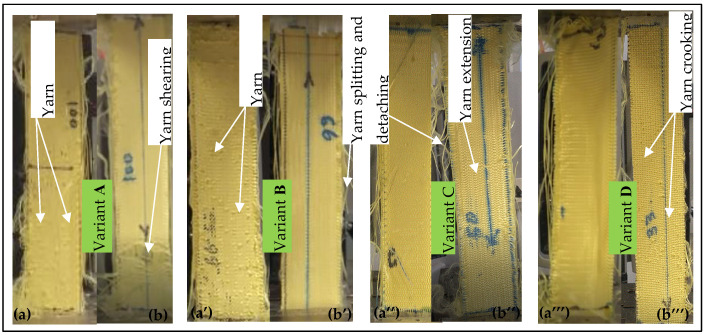
Photographs of 3D warp interlock fabrics at tensile failure in the (**a**,**a’**,**a”**,**a’’’**) weft and (**b**,**b’**,**b”**,**b’’’**) warp direction.

In addition, tensile fabric damage to the samples after each test was also observed and analyzed. Figure 12 shows pictures after testing 3D warp interlock fabric samples at tensile failure state in the warp and weft direction.

The damage mechanism of the fabrics was observed at the tensile deformational failure state based on the macroscopic level. The tensile fabric damage of all samples in the weft direction showed a similar trend, mainly from crooking of weft yarns, as shown in Figure 12a,a’,a”,a’’’. The tensile failure mechanism in the warp direction was different due to the different warp yarn system in each fabric.

For example, variant A exhibited shearing of yarns throughout the sample width near the gripping end until failure. Variants B and C showed similar tensile failure with yarn straightening and extension in the warp direction. Variant D showed similar tensile damage to the other samples in the weft direction. This is because variant D comprised more stuffer than binding warp yarn in the warp direction, which brings similar yarn orientation in the fabric to weft yarn. Regardless of yarn ratio in the fabric structure and the testing direction (warp or weft), all samples showed some yarn splitting from the edges followed by separation along the fabric length. Moreover, higher weft yarn undulation (waviness) due to the involvement of more binding warp yarn in the fabric enhances the tensile failure stress in the respective direction. Such yarn crimp during interlacing in the weaving process could bring extra inter-yarn friction between the yarns and enhance the tensile stress at failure. For instance, the tensile stress of variant A (341.40 ± 10 MPa) and variant D (304.94 ± 10.9 MPa) was found to be higher compared to other fabric samples in the weft and warp direction. Variant B (261.34 ± 12.3 MPa, 297.31 ± 9.8 MPa) and variant C (250.84 ± 9.8 MPa, 333.82 ± 12 MPa) showed different tensile stress at failure in the warp and weft direction due to their higher proportion of binding warp yarn. Based on these observations, the warp yarn composition in the warp directions can influence 3D warp interlock fabric’s maximum tensile strength and strain in the warp direction, as well as in the weft direction. For example, variant D, made with more stuffer warp yarn, showed approximately the same maximum tensile stress at failure in the weft and warp directions. Variant A, with a higher proportion of binding warp yarn (100%), showed a great difference in maximum tensile stress and strain in the warp and weft directions.

### 3.3. Fabric Flexural Rigidity Behavior

Apart from tensile and other mechanical properties, it is also important to investigate flexibility properties, which determine drape comfort and handling of textile materials in various applications. As explained earlier, various factors can affect fabric’s bending behaviors, such as material type, fabric structure, areal density, fabric size, etc. In this section, the influence of warp yarn composition in 3D warp interlock p-aramid fabric on its bending behaviors is discussed.

**Figure 13:**
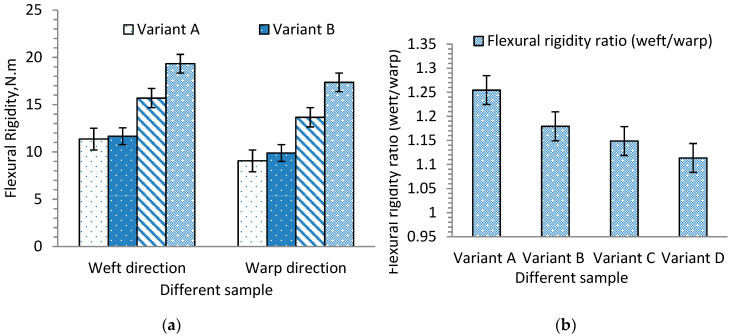
(**a**) Flexural rigidity value of 3D warp interlock fabric samples in weft and warp directions and (**b**) flexural rigidity ratio (weft/warp).

Except for variant A in the warp direction, due to their higher flexural rigidity properties, the other samples were examined for their bending curvature angle (θ) at specific bending length (C). Based on the obtained bending angle and bending length (Figure 5), it is possible to compute the flexural rigidity of each sample using Equation (1). Figure 13a shows the average flexural rigidity values of 3D warp interlock fabrics with warp yarn type composition in the warp and weft directions. It can be seen that fabric with higher stuffer warp yarn composition shows the highest flexural rigidity as compared to fabric with low or no stuffer warp yarn in the warp direction. For example, variant D, made with higher stuffer warp yarn composition, had the highest specific flexural rigidity, followed by variants C and B, whereas variant A, which comprises only binding warp yarn, shows the lowest flexural rigidity. Here, two things are observed and outlined. First, a higher interchange ratio of stuffer yarn greatly affects the bending stiffness of fabric in the longitudinal direction.

For example, it is clearly shown that the fabric sample with 66.7% stuffer warp yarn is about twice as stiff as the samples with no stuffer yarn in the warp direction. It is expected that this trend will be significant and will be reduced for fabrics with less stuffer yarn in their warp composition. The flexural rigidity in the weft direction is also slightly influenced by the warp yarn composition. Even though the weft density and composition were the same for all fabric structures, those in the weft direction were found to be slightly higher than those in the warp direction. This is because weft yarns are generally straighter than warp yarns, which makes a significant difference in bending stiffness. Figure 13b shows the flexural rigidity ratio of each fabric in the weft and warp directions. For example, the flexural rigidity of variant D in the weft direction was found to be higher than that in the warp direction. It can be seen that further reducing the proportion of stuffer yarn in the warp direction helps to reduce the stiffness and results in much lower stiffness in the weft direction. For example, variant A, with no stuffer yarn, shows much less stiffness in the warp direction than the weft direction. Without considering other mechanical properties, increasing the proportion of stuffer yarn in the warp yarn composition of 3D warp interlock fabric is an effective way to obtain higher bending stiffness properties in the warp and weft directions. It is of great interest to compare the two 3D warp interlock fabrics, one with no stuffer yarn and the other with 66.7% stuffer warp yarn. Ideally, the bending stiffness for both should be the same in the weft direction because they both have the same type of yarn, yarn density, and yarn arrangements. However, the difference is due to the orthogonal characteristics of the warp yarn composition, which influences the arrangement of the weft yarn. Thus, fabrics with more stuffer warp yarn have less stress on the weft yarn, which sustains the bending stiffness ability.

## 4. Conclusions

The main aim of the research was to explore and understand the effects of the warp yarn interchange ratio inside the 3D warp interlock p-aramid architectures on its mechanical behavior. For this, three-dimensional (3D) warp interlock p-aramid fabrics with different warp yarn ratios (variant A: 100% binding, 0% stuffer, variant B: 66.7% binding, 33.3% stuffer, variant C: 50% binding, 50% stuffer, and variant D: 33.3% binding, 66.7% stuffer) were fabricated and characterized under quasi-static conditions. Based on the tensile test results, fabric with more stuffer yarn (variant D) revealed higher tensile stress (E; 304.9 MPa) than fabric with less stuffer yarn (variant A, 224.8 MPa, variant B, 261.3 MPa, and variant C, 250.8 MPa). In addition, variant C, with a balanced warp yarn ratio, exhibited two tensile failure points due to the length difference between the two warp yarns. On the contrary, the stress–strain curve in the weft direction showed a linear and progressive trend because the samples had the same weft yarn ratios. Unlike weft direction, variants A (341.4 MPa) and C (333.82 MPa) showed approximately similar tensile stress, with higher values as compared to variants B (297.2 MPa) and C (306.1 MPa) in the warp direction. This is due to the loading effect of warp yarn on weft yarn during the weaving process which showed an influence on tensile strength. Besides, the warp yarn ratio in each variant also affected the maximum tensile strain (εmax) and stress (σmax) at failure in the warp direction. Fabric with a higher binding warp yarn ratio showed higher tensile strain at failure due to the higher crimp properties of the binding warp yarn in the fabric architecture. Variant A showed higher tensile failure strain in the warp (12.88%) than the weft (5.88%) direction, whereas variant D showed approximately similar tensile failure strain in both directions (4.88%) due to the similar undulation of warp and weft yarns. Unlike the warp direction, variants A and B failed at a maximum tensile strain (εmax) of around 5.88% and 7.8% and variables C and D failed at 7.55% and 4.8% respectively, in the weft direction. In addition, variant D (with more stuffer warp yarn) had the highest flexural rigidity (17.36 N.m) compared to fabric with little or no stuffer warp yarn (variant C, 13.66 N.m, variant B, 9.88 N.m, and variant A, 9.05 N.m) in the warp direction. Besides, the flexural rigidity in the weft direction was influenced by the warp yarn ratio and was higher than the respective warp direction due to the smaller waviness properties compared to warp yarns. For example, the fabric with 66.7% stuffer and 33% binding warp yarn (variant D) was about twice as stiff as the ones with no stuffer warp yarn (variant A) in the warp direction.

## Figures and Tables

**Table 1:** Tensile stress and strain at failure for binding and stuffer warp yarns.

	**Binding Warp Direction**		**Stuffer Warp Direction**	
**Sample**	**Tensile Stress, MPa**	**Strain at Failure, %**	**Tensile Stress, MPa**	**Strain at Failure, %**
Variant A	191.11 ± 6.9	5.80 ± 0.9	-	-
Variant B	212.04 ± 7.6	5.56 ± 1.1	222.10 ± 10.1	5.22 ± 0.2
Variant C	188.18 ± 7.9	6.01 ± 0.76	212.73 ± 9.6	5.13 ± 0.11
Variant D	227.9 ± 10.9	5.73 ± 0.6	237.60 ± 12.1	5.76 ± 0.46

**Table 2:** Tensile stress and strain at failure for weft layer yarns in developed variants.

	Variant A		Variant B		Variant C		Variant D	
Weft Layer	Tensile Stress, MPa	Strain at Failure, %	Tensile Stress, MPa	Strain at Failure, %	Tensile Stress, MPa	Strain at Failure, %	Tensile Stress, MPa	Strain at Failure, %
layer 1	216.5 ± 10.9	5.3 ± 0.6	263.5 ± 11.6	5.9 ± 0.5	231.5 ± 11.9	5.8 ± 0.6	233.1 ± 6.9	5.6 ± 0.7
layer 2	217.1 ± 9.9	5.3 ± 0.7	217.0 ± 9.2	5.3 ± 0.9	231.8 ± 10.2	6.0 ± 0.9	231.9 ± 8.9	5.5 ± 0.8
layer 3	224.8 ± 11.2	5.3 ± 0.6	225.9 ± 10.1	5.4 ± 0.9	233.3 ± 12.4	5.5 ± 0.7	231.7 ± 11.1	5.7 ± 1.1
layer 4	211.1 ± 8.9	5.4 ± 0.68	263.0 ± 10.59	5.8 ± 0.6	237.6 ± 10.4	5.8 ± 1.0	218.2 ± 10.9	5.5 ± 1.0
layer 5	214.2 ± 7.2	5.5 ± 0.8	251.6 ± 9.8	6.1 ± 0.9	217.6 ± 8.9	5.5 ± 0.8	211.7 ± 6.9	5.4 ± 0.7

**Table 3:** Tensile stress and strain at failure for variants.

	Warp Direction		Weft Direction	
Sample	Tensile Stress, MPa	Strain at Failure, %	Tensile Stress, MPa	Strain at Failure, %
Variant A	224.80 ± 11.2	12.88 ± 1.1	341.40 ± 10	5.88 ± 0.68
Variant B	261.34 ± 12.3	6.97 ± 0.67	297.31 ± 9.8	7.38 ± 0.9
Variant C	250.84 ± 9.8	8.97 ± 0.7	333.82 ± 12	7.55 ± 0.69
Variant D	304.94 ± 10.9	4.89 ± 0.64	306.10 ± 8.2	4.88 ± 0.76
